# Sources of mercury in deep-sea sediments of the Mediterranean Sea as revealed by mercury stable isotopes

**DOI:** 10.1038/s41598-019-48061-z

**Published:** 2019-08-12

**Authors:** Nives Ogrinc, Holger Hintelmann, Jože Kotnik, Milena Horvat, Nicola Pirrone

**Affiliations:** 1 Department of Environmental Sciences, Jožef Stefan Institute, Ljubljana, Slovenia; 2 Water Quality Centre, Trent University, Peterborough, Canada; 3 CNR-Institute of Atmospheric Pollution Research, Rome, Italy

**Keywords:** Element cycles, Environmental sciences

## Abstract

Mercury (Hg) and its stable isotope composition were used to determine the sources of Hg in deep-sea sediments of the Mediterranean Sea. Surface and down-core sediment δ^202^Hg values varied widely between −2.30 and +0.78‰, showed consistently positive values for mass independent fractionation of odd Hg isotopes (with average values of Δ^199^Hg = +0.10 ± 0.04‰ and Δ^201^Hg = +0.04 ± 0.02‰) and near-zero Δ^200^Hg values, indicating either multiple Hg sources or a combination of different Hg isotope fractionating processes before and after sediment deposition. Both mass-dependent and mass-independent fractionation processes influence the isotopic composition of Hg in the Mediterranean Sea. Positive Δ^199^Hg values are likely the result of enhanced Hg^2+^ photoreduction in the Mediterranean water column before incorporation of Hg into sediments, while mass-dependent fractionation decreases δ^202^Hg values due to kinetic isotope fractionation during deposition and mobilization. An isotope mixing model based on mass-dependent and mass-independent fractionation (δ^202^Hg and Δ^199^Hg) suggests at least three primary Hg sources of atmospheric deposition in the surface sediments: urban, industrial and global precipitation-derived. Industry is the main source of Hg in Algerian and Western Basin surface sediments and at two sites in the Adriatic Sea, while the urban contribution is most prominent at the Strait of Otranto (MS3) and in Adriatic surface sediments. The contribution from precipitation ranged from 10% in Algerian to 37% in W Basin sediments. Overall, results suggest that atmospheric Hg deposition to Mediterranean surface sediments is dominated by gaseous elemental mercury (58 ± 11%) rather than wet deposition.

## Introduction

The oceans play a very important role in global mercury (Hg) cycling. Once deposited, Hg can be reduced to elemental Hg and volatilized back into the atmosphere, accounting for as much as half of the mercury present in the global atmosphere. The Mediterranean Sea is of particular concern since it is impacted by multiple Hg pollution sources. Natural Hg is derived from tectonic activities and volcanic emissions, while anthropogenic sources of Hg originate from industrial and mining activities. In order to understand Hg cycling in water and sediments of the Mediterranean Sea, as well as the relative contributions of Hg from natural and anthropogenic sources, detailed measurements and modelling studies were conducted^1,2^. The results suggested that the main Hg sources and sink is exchange with the atmosphere; evasion contributes roughly 70% to the Hg output and deposition about 45% to the input. Sediment exchange is the next most important mechanism, contributing 25–30% to both inputs and outputs, while river input contributes around 14%. All other sources and sinks are less important with regard to the overall Hg mass balance. To further support these findings, isotope fractionation patterns can provide a useful tool to understand and quantify Hg sources, sinks and transformations. Research into Hg stable isotope biogeochemistry is rapidly providing new insights into the behaviour of Hg^3^. The seven naturally occurring stable isotopes (^196^Hg, ^198^Hg, ^199^Hg, ^200^Hg, ^201^Hg, ^202^Hg and ^204^Hg) exhibit both mass-dependent (MDF) and large mass-independent fractionation (MIF) (range of >6‰ for both). MIF signatures often indicate specific chemical processes, such as photochemical reduction, which greatly increases the usefulness of Hg isotope ratio determinations^4^. Typically, photoreactions produce positive Δ^199^Hg in Hg remaining in the aqueous phase and negative Δ^199^Hg in the atmosphere^5–7^, which is recorded in lichens and other plant samples^8^. However, precipitation is often characterized by positive Δ^199^Hg^9–11^. In a similar fashion, MDF can improve our understanding of the processes that control Hg distribution and bioaccumulation.

Marine sedimentary Hg is often dominated by Hg of geogenic origin, but have also shown to retain anthropogenic contributions in surface layers^12,13^, while aqueous Hg is more exposed to photochemical and microbial reactions. Foucher *et al*.^12^, demonstrated that there is a well-resolved difference between the Hg isotopic composition of Adriatic Sea sediment (depleted in ^202^Hg) and Hg in cinnabar derived originally from the Idrija region (enriched in ^202^Hg), which drains into the Gulf of Trieste. On the other hand, no difference in Hg isotopic composition was observed between sapropels and historic sediments despite the 6-fold difference in Hg concentration in deep-sea Tyrrhenian Basin sediments^13^. This similarity may suggest that the marine sediments reflect the Hg isotopic composition of ambient seawater, or conversely may indicate that the isotopic composition of Hg in the ocean reflects that of Hg in the upper crust. Atmospheric deposition is the largest source of Hg to the global oceans. Pre-industrial atmospheric Hg originated primarily from volcanic and hydrothermal emissions. Recent measurements of volcanic and sedimentary deposits in California showed Hg isotopic compositions close to values reported for Pleistocene Mediterranean seawater^14^.

The aim of the present study is to investigate potential sources of Hg in Mediterranean deep-sea and Adriatic sediments. To our knowledge only a few studies report stable isotope compositions of Hg in deep-sea sediments and most of them were conducted close to estuaries or coastal region^15–18^. Ogrinc *et al*.^19^ previously determined concentrations of Hg in sediments that were further characterized by C and N concentrations and isotopic composition. In this study, stable isotope ratios of Hg were utilized to better understand Hg sources and processing pathways in marine deep-sea sediments including depth profiles. Specifically, we suggest that the determination of δ^202^Hg, and Δ^199^Hg values in sediments can be used to develop a triple mixing model that provides estimates of the relative amounts of Hg derived from urban, industry and global atmospheric deposition.

## Results and Discussion

### Hg and its isotope composition in surface sediments

**Figure 1:**
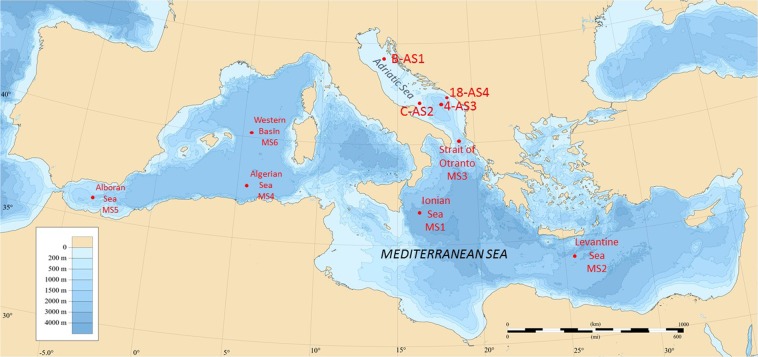
Sediment sampling sites in the Mediterranean and Adriatic Sea.

Organic carbon content (OC), concentrations and stable isotope data of Hg at selected locations in deep-sea sediments in Mediterranean and Adriatic Sea (Fig. 1) are collected in Table 1. If we take into account sedimentation rates between 0.012 and 0.024 cm yr^−1^ ^20^ determined in the Western and 0.003 cm yr^−1^ in the Eastern Basin^21^, only the first few cm correspond to the industrial period and have therefore the potential to show an influence of anthropogenic sources. The spatial variation of total Hg (HgT) in surface sediments ranged from 14 (MS2) to 153 ng g^−1^ (MS3 and MS4) in the Mediterranean and Adriatic Seas. HgT concentrations were normalized to OC content, because Hg often correlates closely with OC phases in sediments^22^. The degree of Hg enrichment relative to background values was defined as the enrichment factor (EF = (HgT/OC)_sample_/(HgT/OC)_background_)_,_ where the HgT background concentrations were taken from the average determined for the Levantine Sea sediments (MS2). The highest EF factor was observed at the surface ranging from 1.1 to 2.7 at AS1 and MS3, respectively. The EF > 1.5 at the surface sediments was observed at MS3, MS4, MS6 and AS3 indicating minor enrichment with Hg.

**Table 1:** Organic carbon content (OC), concentrations and stable isotope data of Hg together with the estimated contribution of different sources (urban (Urb), industrial (Ind), global precipitation (Glob)) with the estimated uncertainty in deep-sea sediments in Mediterranean and Adriatic Sea

Site ID	Core depth	Hg	OC	δ^199^Hg	δ^200^Hg	δ^201^Hg	δ^202^Hg	Δ^199^Hg	Δ^200^Hg	Δ^201^Hg	Δ^199^Hg/Δ^201^Hg	Urb	Ind	Glob
	cm	ng g^−1^	wt.%	‰	‰	‰	‰	‰	‰	‰		%	%	%
Ionian Sea MS1	0–1	41.7	0.37	−0.24	−0.58	−0.97	−1.28	0.09	0.07	0.00	0.00	32 (±14)	41 (±24)	27 (±22)
	1–5	50.4	0.27	−0.33	−0.75	−1.23	−1.62	0.08	0.06	−0.02	−4.45	—	—	—
	>5	66.7	0.48	−0.44	−1.04	−1.59	−2.18	0.11	0.05	0.05	2.00	—	—	—
Levantine Sea MS2	0–1	14.9	0.27	−0.18	−0.51	−0.82	−1.15	0.11	0.07	0.05	2.31	25 (±14)	43 (±25)	32 (±32)
	1–2	27.7	0.23	−0.21	−0.56	−0.84	−1.13	0.08	0.01	0.02	4.61	—	—	—
	2–3	28.7	0.17	−0.18	−0.49	−0.84	−1.01	0.07	0.02	−0.08	−0.87	—	—	—
	>3	23.2	0.17	−0.20	−0.53	−0.78	−1.07	0.07	0.01	0.03	2.66	—	—	—
Strait of Otranto MS3	0–1	153	0.59	−0.49	−1.13	−1.66	−2.30	0.09	0.03	0.08	1.23	88 (±6)	2 (±5)	10 (±7)
	1−5	48.3	0.47	−0.30	−0.74	−1.04	−1.51	0.08	0.02	0.09	0.85	—	—	—
	5–7	46.3	0.74	−0.32	−0.85	−1.24	−1.68	0.10	−0.01	0.02	4.59	—	—	—
	7–10	51.6	0.52	−0.43	−1.04	−1.56	−2.13	0.11	0.03	0.03	3.14	—	—	—
	>10	44.1	0.67	−0.41	−1.10	−1.68	−2.24	0.15	0.03	0.00	0.00	—	—	—
Algerian Sea MS4	0–1	153	0.63	−0.25	−0.50	−0.80	−0.95	0.03	0.05	0.03	0.98	19 (±15)	62 (±23)	19 (±23)
	1–4	84.5	0.89	−0.24	−0.61	−0.92	−1.33	0.10	0.05	0.08	1.16	—	—	—
	4–8	144	0.44	−0.23	−0.53	−0.82	−1.16	0.06	0.05	0.06	1.07	—	—	—
	>8	67.6	1.03	−0.29	−0.67	−1.01	−1.35	0.05	0.00	0.01	8.75	—	—	—
Alboran Sea MS5	0–1	115	0.86	−0.17	−0.55	−0.89	−1.22	0.13	0.06	0.03	4.24	29 (±14)	36 (±24)	35 (±24)
	1–4	92.0	1.00	−0.48	−1.11	−1.72	−2.28	0.10	0.03	−0.01	−11.08	—	—	—
	4–8	82.3	0.77	−0.43	−1.09	−1.66	−2.23	0.14	0.03	0.02	6.84	—	—	—
	>8	55.3	0.79	−0.43	−1.03	−1.55	−2.12	0.11	0.03	0.04	2.78	—	—	—
Western basin MS6	0–1	98.6	0.55	−0.08	−0.39	−0.52	−0.78	0.11	0.00	0.06	1.77	10 (±11)	53 (±26)	37 (±27)
	1–4	65.1	0.33	−0.12	−0.38	−0.60	−0.82	0.09	0.03	0.02	4.65	—	—	—
	4–8	60.6	0.29	−0.19	−0.55	−0.83	−1.16	0.11	0.03	0.05	2.33	—	—	—
	>8	34.7	0.99	−0.22	−0.59	−0.94	−1.20	0.08	0.02	−0.03	−2.66	—	—	—
Adriatic sites														
AS1	0–1	41.7	0.35	−0.32	−0.87	−1.36	−1.88	0.15	0.08	0.05	3.08	65 (±9)	8 (±13)	27 (±13)
AS2	0–1	45.6	0.41	−0.11	−0.37	−0.60	−0.85	0.10	0.06	0.04	2.82	13 (±12)	53 (±24)	25 (±24)
AS3	0–1	65.6	0.55	−0.20	−0.51	−0.77	−1.07	0.07	0.03	0.03	2.15	22 (±15)	53 (±24)	25 (±24)
AS4	0–1	46.4	0.38	−0.32	−0.77	−1.14	−1.61	0.09	0.04	0.07	1.20	51 (±12)	25 (±20)	24 (±18)

**Figure 2:**
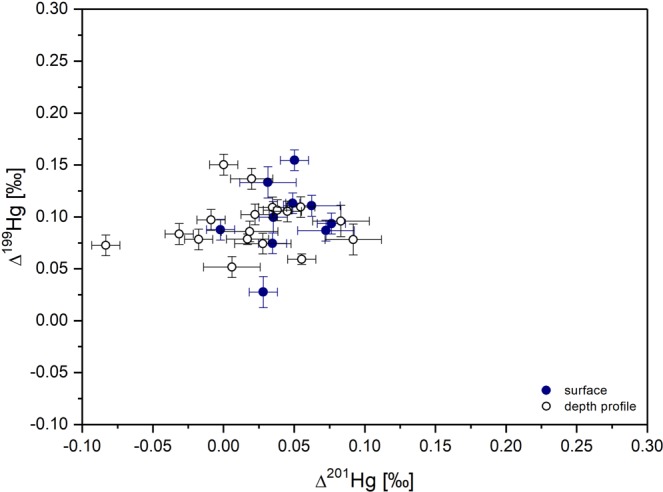
Δ^199^Hg versus Δ^201^Hg in deep-sea Mediterranean and Adriatic sediments. Error bars on samples in the main panel indicate one standard deviation of the analytical uncertainty.

Measured δ^202^Hg values in surface sediments were variable and ranged from −2.30 at MS3 to −0.78‰ at MS6. The measured δ^202^Hg values fall between the average δ^202^Hg of −0.76 ± 0.16‰ established for background Mediterranean Sea sediment^14^ and the average δ^202^Hg of −2.13 ± 0.46‰ for background Adriatic Sea sediments^12^. Positive MIF of odd Hg isotopes (^199^Hg and ^201^Hg) was observed in surface sediments with average values of Δ^199^Hg = +0.10 ± 0.04‰ and Δ^201^Hg = +0.04 ± 0.02‰ (Fig. 2). Similar Δ^199^Hg values of +0.10‰ were reported for modern Pacific deep-sea sediments^23^, Archean marine black shales (+0.15‰^24^,) and the South China Sea (mean, +0.35 ± 0.09‰^15^,). The observed positive MIF in sediments could be a result of Hg settling from the water column, considering that photoreduction of Hg(II) typically leads to ^199^Hg enrichment in the aqueous phase^6^. Another possible explanation for the observed positive Δ^199^Hg values could be atmospheric Hg deposition. A recent mass balance for the Mediterranean Sea suggested that as much as 45% of the Hg input is by atmospheric deposition^2^. This would be in accordance with global Hg rainfall observations, which display positive Δ^199^Hg (+0.37 ± 0.25‰, 1σ, n = 105)^11^. Positive ∆^199^Hg values (0 to +1.0‰) have been reported for precipitation collected from different sites of the world^9,25–28^. In general, negative Δ^199^Hg values were reported in gaseous elemental Hg (GEM), while positive Δ^199^Hg values were reported in gaseous oxidized Hg (Hg^2+^) and particulate/aerosol-bound Hg (Hg_p_) species^7,26,29^. In this study, no MIF was observed for ^200^Hg, which is consistent with previous studies in ocean sediments^12,14,22^. Although ∆^200^Hg values different from 0 was frequently reported for atmospheric samples, ∆^200^Hg values of 0 in sediments might be explained by the mixing of gaseous elemental mercury (GEM, with negative Δ^200^Hg values) with oxidized atmospheric Hg species in precipitation (with positive Δ^200^Hg values). A Δ^200^Hg isotopic mass balance using the above global rainfall end-member (+0.18 ± 0.15‰, 1σ, n = 105) and global GEM end-member (Δ^200^Hg = −0.05 ± 0.04‰, 1σ, n = 69) would suggest that 59 ± 11% (1σ) of Mediterranean surface sediment Hg was ultimately derived from GEM dry deposition, resulting in a net zero Δ^200^Hg.

The data from the Levantine basin (MS2) may represent the background of Mediterranean deep-sea sediments, having HgT concentrations as low as 14 ng g^−1^. No influence of direct continental and/or volcanic sources could be identified, which is further supported by the sediment δ^13^C_OC_ value of −21.6‰^19^. The large amount of Ca found at MS2 reflects the presence of high concentrations of CaCO_3_, normally associated with biogenic pelagic deep-sea sediments (foraminiferal oozes).

At MS4, MS5 and AS2 the more positive δ^202^Hg values were associated with higher Hg concentrations, which may suggest a recent Hg source. Other studies suggested that industrial sources exhibit δ^202^Hg values closer to zero (−1 to 0‰) and insignificant MIF (Δ^199^Hg ∼ 0‰)^30,31^. The lowest δ^202^Hg value of −2.30‰ was observed at MS3 and is more similar to δ^202^Hg values (δ^202^Hg: −2 to −3‰) found in precipitation and atmospheric samples impacted by anthropogenic Hg emissions^12,15^. A recent study indicated that Hg in sediment at the Strait of Otranto (MS3) might originate from particulate Hg transported by the water currents from the Adriatic Sea^32^.

More negative δ^202^Hg values in the study area may also be explained by inputs from two others possible sources often characterized by low δ^202^Hg: (1) most terrestrial samples (soils, foliage, litter, lichens) display negative δ^202^Hg (−1.3 ± 0.8‰, 1 σ, n = 162) and Δ^199^Hg (−0.2 ± 0.2‰, 1 σ, n = 163), and insignificant Δ^200^Hg (0.03 ± 0.04‰, 1 σ, n = 119)^7,11,33^; (2) atmospheric Hg emitted from anthropogenic sources generally has very low δ^202^Hg values. For instance, precipitations from urban-industrial regions in China have shown highly negative δ^202^Hg values as low as −4.27‰^27^, while total gaseous Hg collected in the Wanshan Hg mine (southwest China) showed negative δ^202^Hg ranging from −2.32 to −1.85‰^34^. Gaseous Hg° collected near a coal-fired power plant (Grand Bay, U.S.A.) displayed negative δ^202^Hg from −3.88 to −0.33‰^29^. Precipitation collected close to a coal-fired utility boiler (Crystal River, U.S.A.) also displayed large negative δ^202^Hg values (mean, −2.56‰; n = 28)^26^. Sun *et al*.^35^ estimated the modern-day mean δ^202^Hg and Δ^199^Hg values for bulk coal emissions of −1.2 ± 0.5‰ (1 SD) and 0.05 ± 0.06‰ (1 SD). It should be mentioned that also in the Mediterranean Basin coal and oil combustion represents an important anthropogenic source of Hg.

### Hg and its isotope composition in sediment cores

**Figure 3:**
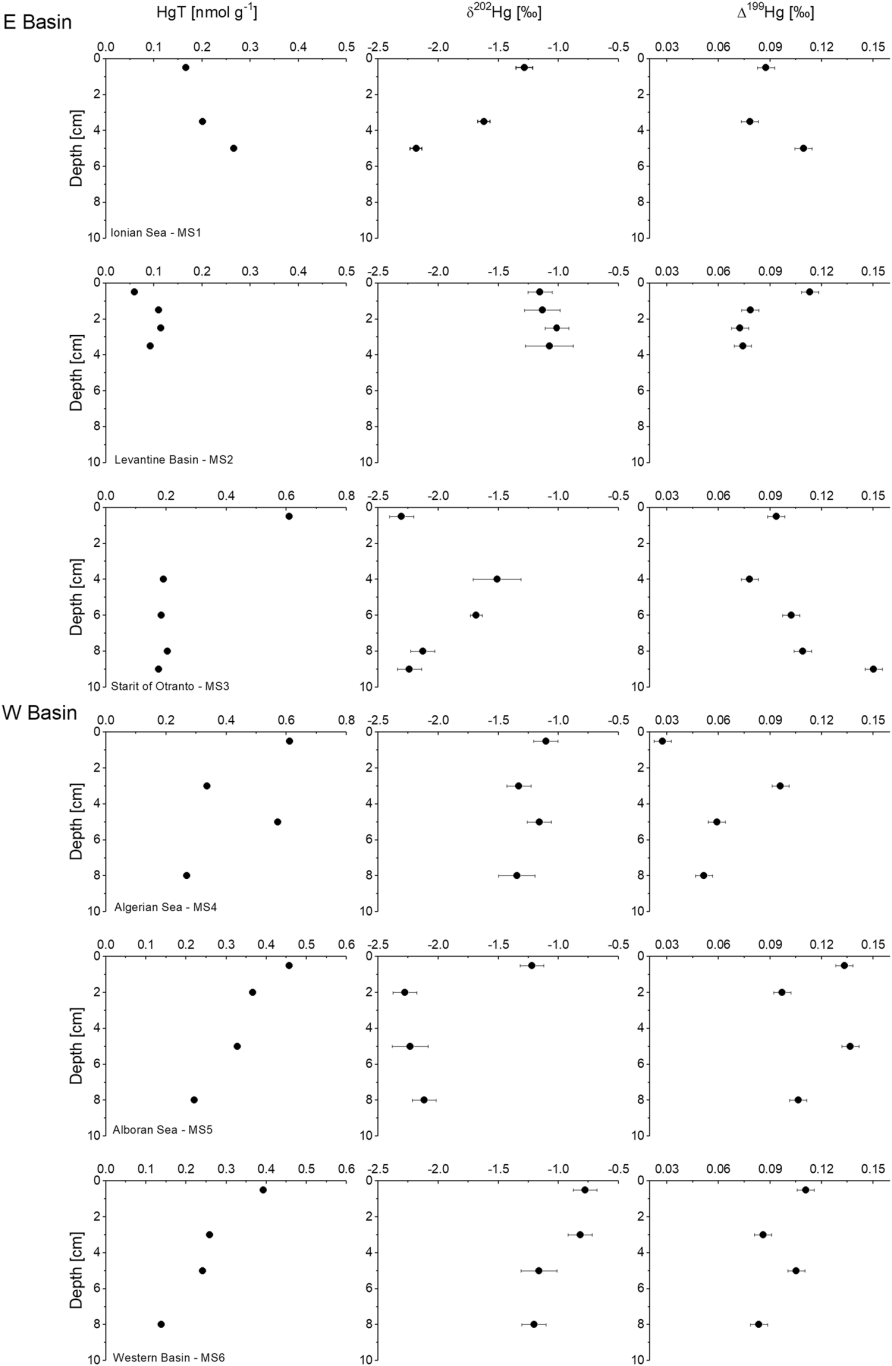
Depth profiles of HgT concentrations, δ^202^Hg and Δ^199^Hg in deep-sea sediments at different locations of the Mediterranean Sea. Error bars on samples indicate one standard deviation of the analytical uncertainty.

The concentrations of HgT and values of δ^202^Hg in sediment cores are presented in Fig. 3. Increasing concentrations of HgT were occasionally determined deeper in the cores, which may reflect changes or redistribution during diagenetic processes. In the Eastern Basin, the origin of the deeper HgT concentration variations could also be a consequence of natural variability caused by sea-level fluctuations or seismic activity. Down-core δ^202^Hg values do not show a clear pattern and were site specific implying either multiple sources, or varying amounts of microbial Hg reduction and loss, or a combination of both (Fig. 3).

MS2 sediments show δ^202^Hg and Δ^199^Hg values of −1.09 ± 0.06‰ and 0.08 ± 0.02‰, respectively, similar to previously published data for the Tyrrhenian Sea (average δ^202^Hg of −0.76 ± 0.16‰; Δ^199^Hg = 0.11 ± 0.03‰)^14^, pre-industrial Baltic Sea sediments (average δ^202^Hg = −1.04 ± 0.18‰ and Δ^199^Hg = 0.13 ± 0.04‰)^36^ and background values (δ^202^Hg: ∼ −1.0‰; Δ^199^Hg: ~ +0.2‰) in coastal seas^37,38^. No significant change in δ^202^Hg and Δ^199^Hg values with depth was observed at MS2.

The HgT concentrations at MS1 show an increase with depth together with a decrease in δ^202^Hg from −1.28 to −2.18‰ (Fig. 3). Assuming a sedimentation rate of 0.003 cm yr^−1^ for the Eastern Basin^21^, sediments at >5 cm depths likely correspond to AD 300 or older. Thus, these layers are pre-modern sediments and if the isotope signature was related to sources, the Hg may only originate from volcanic and/or hydrothermal emissions. More specifically, the low δ^202^Hg values could possibly reflect the isotope composition of Hg in ash originating from intensive volcano activity. This interpretation is further supported by previous studies indicating that these sediments contain the markers (tephroanalysis) of well-known historical eruptions (Pompei, AD 79, Pollena, AD 472, Ischia, AD 1301, dates that are all captured within the top 5 cm)^39^. A recent study of volcanic and sedimentary rock from California exhibited higher δ^202^Hg values ranging from −1.0 to −0.6‰ and Δ^201^Hg values of ∼ 0‰^15^, while studies performed at an active volcano in Italy showed high variability in δ^202^Hg values ranging from −1.74 to −0.11‰^40,41^. However, diagenetic mobilization of anthropogenic Hg downcore could also not be excluded. Mobilization of part of the Hg adsorbed to the sediment is accompanied by fractionation resulting in the enrichment of the mobilized Hg with light isotopes (MDF). At this point, we can only speculate about the atmospheric Hg isotopic composition prior to human perturbation, since anthropogenic emissions are now about twice the natural emissions^42^. In addition, processes that fractionate Hg isotopes in the atmosphere are poorly characterized. Nevertheless, the observed positive Δ^199^Hg data are broadly consistent with Hg as a product of photoreduction^5,43^.

In all other cores (MS3 to MS6) a decrease in Hg concentrations was observed with depth, while no clear pattern in δ^202^Hg values was discernible (Fig. 3). Low δ^202^Hg values of ∼ −2‰ were observed also deeper in the sediments at MS3 and MS5 (Fig. 3). Environmental processes such as microbial reduction^44^, photoreduction^3,44^, photodemethylation^3,44^, methylation^45^ and evasion^46,47^ always show a preferential loss of the lighter Hg isotope leaving the study system and the residual fraction enriched in the heavier isotope. On the other hand, mobilization causes the enrichment of Hg with the light isotope and cannot be excluded. The diagenetic remobilization of Hg from deeper layers is possible in areas characterized by lower sedimentation rates^48^ and may affect the distribution of Hg particularly in the Eastern Basin. An effective role in sequestration of Hg, such as adsorption on Fe and Mn oxyhydroxide was observed in Mediterranean sediments^15^, which could also change MDF. Laboratory-based studies showed an MDF shift of −0.4‰ as Hg absorbs on goethite^33^.

**Figure 4:**
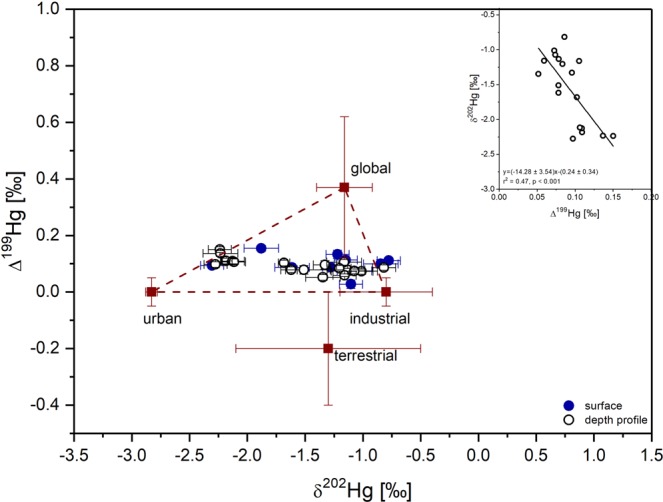
The relationships between δ^202^Hg and Δ^199^Hg in Mediterranean and Adriatic sediments and end-members used for the triple-mixing model. Error bars on samples in the main panel indicate one standard deviation of the analytical uncertainty. The inset of panel shows the relationships between δ^202^Hg and Δ^199^Hg values in core deep-sea Mediterranean sediments.

It should be also mentioned that more negative δ^202^Hg values are usually associated with the highest ∆^199^Hg values with a significant negative correlation of −14.28 (P < 0.001) (Fig. 4). Laboratory experiments on Hg^2+^ photoreduction revealed δ^202^Hg/∆^199^Hg of 0.83^49^, while for Tibetan Lakes the ratio was much higher with δ^202^Hg/∆^199^Hg = 8.88 and 5.75^50^. Further no correlation between ∆^199^Hg and HgT (P > 0.05), was observed, which may suggest that Mediterranean sediments may be more influenced by in-ocean processes. The increase of Δ^199^Hg is likely the result of enhanced Hg^2+^ photoreduction in the Mediterranean water column before Hg is incorporated into sediments. For example, the importance of atmospheric transformation processes occurring in the marine boundary layer (MBL) may vary due to varying meteorological and climatic conditions in the Mediterranean Basin (i.e. warmer climate, high temperature and strong solar radiation)^1^, having a more or less pronounced effect on the fractionation of Hg isotopes. Possibly, (photo)processes created initially positive MIF and MDF, but subsequent stronger MDF-only reactions led to overall negative δ^202^Hg values, while conserving the initial Δ^199^Hg signature. This MDF fractionation can occur during settling or after sedimentation. Until now, the only known process generating a negative shift in δ^202^Hg is photoreduction of Hg complexed by thiols or Fe, Mn oxyhydroxides. Significant δ^202^Hg shifts are more likely to occur when only very small fractions of Hg are adsorbed relative to the total Hg in the system, which is actually the case in Mediterranean Sea^1,2^. Thus, further research on water column Hg processes in the Mediterranean Sea are needed to better understand the variations of Hg isotopes in this study.

### Source apportionment model for mediterranean and adriatic surface sediments

Binary and/or triple mixing models have previously been employed to estimate the relative contribution of sources of Hg^12,13,16,31,37,51–54^. In this study, we propose that the relationship between concentrations and stable isotope data of Hg in Mediterranean and Adriatic surface sediments can be modelled using three sources, all three derived as emissions from the atmosphere (Fig. 4): industrial Hg (δ^202^Hg, −0.40‰; Δ^199^Hg, 0.05‰), urban Hg (δ^202^Hg, −2.23‰; Δ^199^Hg, 0.05‰), and global precipitation Hg (δ^202^Hg, −0.56 ± 0.24‰; Δ^199^Hg, +0.42 ± 0.25‰). We applied an adsorption shift of −0.6‰^33^ for our end-members, as a result of aquatic sediments particle adsorption during deposition, overall deposition shift of 0.4‰ and 0.05‰ for δ^202^Hg and Δ^199^Hg, respectively, as determined by Archer and Blum^55^. Fig. 4 also shows the global terrestrial end-member with δ^202^Hg of −1.3 ± 0.8‰ (1σ, n = 162) and Δ^199^Hg of −0.2 ± 0.2‰ (1σ, n = 163)^7,11,56^. It is evident that the terrestrial input was not a good fit as a potential source of Hg, which is also supported by recent mass balance for the Mediterranean Sea, estimating that rivers contribute only 14% of the total Hg inputs^2^. We are aware that these values may only be crude approximations at this point and the composition of Hg deposited onto sediments is likely a complex end product of a series of processes and pathways including photoreduction and photodemethylation, which influence Δ^199^Hg values post deposition/input to oceans.

Taking into account all three end-members the following ternary mixing model was used:

1 $${{\rm{\delta }}}^{{\rm{202}}}{{\rm{Hg}}}_{{\rm{glob}}}{{\rm{F}}}_{{\rm{glob}}}+{{\rm{\delta }}}^{{\rm{202}}}{{\rm{Hg}}}_{{\rm{ind}}}{{\rm{F}}}_{{\rm{ind}}}+{{\rm{\delta }}}^{{\rm{202}}}{{\rm{Hg}}}_{{\rm{urb}}}{{\rm{F}}}_{{\rm{urb}}}={{\rm{\delta }}}^{{\rm{202}}}{{\rm{Hg}}}_{{\rm{sam}}}$$

2 $${{\rm{\Delta }}}^{{\rm{199}}}{{\rm{Hg}}}_{{\rm{glob}}}{{\rm{F}}}_{{\rm{glob}}}+{{\rm{\Delta }}}^{{\rm{199}}}{{\rm{Hg}}}_{{\rm{ind}}}{{\rm{F}}}_{{\rm{ind}}}+{{\rm{\Delta }}}^{{\rm{199}}}{{\rm{Hg}}}_{{\rm{urb}}}{{\rm{F}}}_{{\rm{urb}}}={{\rm{\Delta }}}^{{\rm{199}}}{{\rm{Hg}}}_{{\rm{sam}}}$$

3 $${{\rm{F}}}_{{\rm{glob}}}+{{\rm{F}}}_{{\rm{ind}}}+{{\rm{F}}}_{{\rm{urb}}}={\rm{1}}$$

where subscripts “glob”, “ind”, “urb” and “sam” are related to global precipitation, industrial, urban and sample, respectively. The uncertainty of the extracted F parameters was determined using stochastic Monte Carlo simulations. For each experimental parameter δ^202^Hg_*x*_ and Δ^199^Hg_*x*_ a normal distribution was created characterized by its mean and standard deviation values. For all parameters (i.e. δ^202^Hg_glob_, δ^202^Hg_ind_, δ^202^Hg_urb_, δ^202^Hg_samp_, Δ^199^Hg_glob_, …) a random value was later calculated from the corresponding distribution. These values were used to solve the equations (1–3). The results of different source contributions with standard deviation of calculated F numbers are presented in Table 1. We also estimate the strongest influence of experimental parameters on the extracted F parameters and found out that δ^202^Hg_urb_ and Δ^199^Hg_urb_ have the highest effect on all calculations. The model suggests that Hg pollution from the industry represents the main source of Hg at the surface at MS4, MS6, AS2 and AS3, while the urban contribution was the highest at MS3 and AS1. The contribution from global precipitation ranged from 10% at MS3 to 37% at MS6.

We acknowledge the uncertainty of this model, as exact end-members have not been directly measured. However, the results represent the first rough estimates of the proposed sources to overall Hg distribution in surface deep-sea sediments in the Mediterranean and Adriatic Sea. It still contains several simplifications that require refinement in further studies. For example, the end members of the model are currently based only on a small dataset, relative to the size of the study area. Especially the high uncertainty for Hg isotope signatures in global precipitation should be addressed. Consequently, the model is to be used with caution, especially in the open ocean where secondary fractionation processes may impact the original source signatures. Addressing these uncertainties will strengthen the use of Hg isotope ratios as a tool to attribute sources of Hg in a complex system such as the Mediterranean Sea and eventually also other world oceans.

## Materials and Methods

### Study area and sampling

A site description and sampling protocol employed in collection of the samples studied here are provided in Ogrinc *et al*.^19^. Briefly, sediment samples were collected with a box corer from six sites during an oceanographic sampling campaign aboard the Italian research vessel Urania in August 2003 in the Eastern and Western basins of the Mediterranean and from four sites in October-November 2004. In addition, surface sediments were collected from four Adriatic sites, while profiles of up to 10 cm depths were taken from Mediterranean Sea locations. Sampling locations are presented in Fig. 1.

### Organic carbon (OC) content

OC content in sediments was determined by a Carlo Erba elemental analyzer (model EA 1108) after acidification with 1 N HCl to remove carbonate material and is expressed in wt.%. The precision of measurements was ± 3%.

### Mercury isotope ratio analysis

Typically, 0.2 g of dried sediment was digested with 10 ml of a concentrated acids mixture (HNO_3_/HCl, 7:3 v/v) in open glass vessels. Digestion was performed on a hot plate at 120–140 °C for approx. 6 h and then diluted to 40 ml with Milli-Q water. Where necessary, the mass of sediment was increased to achieve a final concentration of Hg of at least 1 µg l^−1^. Concentrations for the bracketing standard (NIST 3133) were adjusted to match Hg concentrations in sediment digests to within 10%. Hg isotopic compositions were determined using a continuous Hg° vapour generation method and analysis using a multiple-collector inductively coupled plasma mass spectrometer (MC−ICP/MS) equipped with nine Faraday cups (Neptune, Thermo Fisher Scientific, Bremen, Germany). A more detailed description of the overall instrumental setup and analytical conditions used in this study can be found elsewhere^12,57^. Results for Hg isotope ratios are reported as the deviation from a common Hg standard solution (NIST 3133 Hg) using the customary δ-notation expressed in per mil (‰)^58^:

4 $${{\rm{\delta }}}^{{\rm{xxx}}}{\mathrm{Hg}/}^{{\rm{198}}}{\rm{Hg}}=[({{\rm{R}}}_{{\rm{sample}}}{/{\rm{R}}}_{{\rm{standard}}})-{\rm{1}}]\times 1000$$

where R_sample_ is the measured ^xxx^Hg/^198^Hg ratio for the unknown sample, R_standard_ is the mean ^xxx^Hg/^198^Hg ratio of the bracketing δ-zero standard NIST 3133 and ‘xxx’ is the mass number of Hg (199, 200, 201, or 202). The ^202^Hg/^198^Hg isotope pair was selected to describe MDF, while MIF are characterized using the “capital delta” notation (Δ) as follows:

5 $${{\rm{\Delta }}}^{{\rm{xxx}}}{\rm{Hg}}\approx {{\rm{\delta }}}^{{\rm{xxx}}}{\rm{Hg}}-{{\rm{\delta }}}^{{\rm{202}}}{\rm{Hg}}\times {\rm{\beta }}$$

where β is the scale factor of the theoretical MDF law and is equal to 0.2520 for ^199^Hg, 0.5024 for ^200^Hg, and 0.7520 for ^201^Hg^58^. Data uncertainties reported in this study reflect the larger values of either the external precision of the replication of the UM-Almadén standard solution or the measurement uncertainty of repeated sample analysis. The overall measured average and uncertainty for UM-Almadén was δ^202^Hg = −0.52 ± 0.09‰; Δ^199^Hg = −0.01 ± 0.05‰; Δ^200^Hg = 0.00 ± 0.03‰; and Δ^201^Hg = 0.00 ± 0.05‰, for 2σ level. These results agreed well with previous studies^58^.
